# Navigating the AI tide: challenges, opportunities, and future directions for early-career dermatologists

**DOI:** 10.3389/fmed.2025.1684035

**Published:** 2025-10-01

**Authors:** Meng Zhang, Ruiqi Chu, Chunmei Liu, Shengni Zhang, Xiangxiang Ren

**Affiliations:** ^1^Department of Dermatology, Affiliated Hospital of Hebei University, Baoding, Hebei, China; ^2^Department of General Surgery, Affiliated Hospital of Hebei University, Baoding, Hebei, China

**Keywords:** artificial intelligence, dermatologic diagnosis, early-career dermatologists, human-AI collaboration, ethical and legal challenges

## Abstract

Artificial intelligence (AI) has demonstrated diagnostic accuracy comparable to dermatologists in specific tasks (e.g., 92.5% vs. 86.6% for melanoma detection in multicenter trials), while significantly outperforming early-career physicians (15–20% higher accuracy in meta-analyses). This review synthesizes evidence on AI’s transformative impact on dermatology training and practice, addressing critical gaps in ethical frameworks and implementation strategies. We propose a competency-based framework for “AI-augmented dermatology,” advocating for curriculum integration of AI literacy modules, standardized human-AI workflows, and proactive engagement in regulatory processes. Early-career dermatologists must leverage AI as a safety net while strengthening irreplaceable skills in complex decision-making and patient communication to lead dermatology’s AI-integrated future.

## 1 Current capabilities and limitations of AI in dermatologic diagnosis

**Figure 1:**
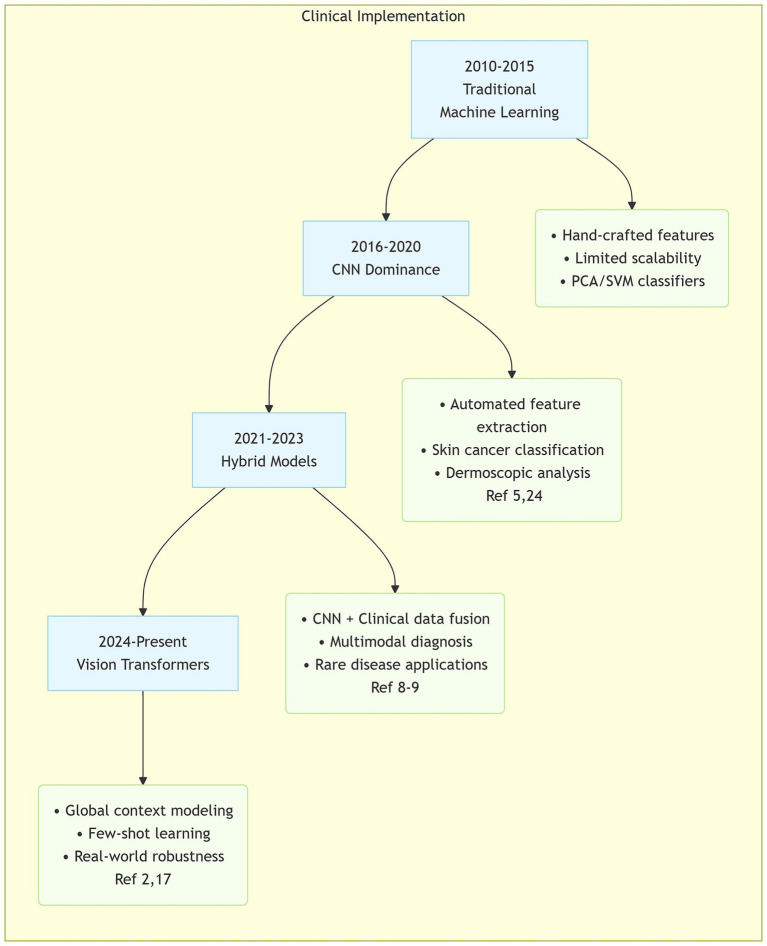
Key technical roadmap of AI in dermatology. Evolutionary pathway of core AI technologies in dermatology applications, highlighting architectural transitions and clinical implementation milestones.

Artificial intelligence (AI), particularly algorithms utilizing Convolutional Neural Networks (CNNs), has demonstrated significant potential in analyzing dermatologic images, including dermoscopic and clinical photographs (Figure 1). A clear understanding of both its diagnostic capabilities and current limitations represents essential knowledge for dermatologists navigating this evolving landscape.

### 1.1 Diagnostic accuracy: comparative assessment with dermatologists

Substantial evidence demonstrates that AI, particularly validated deep learning models, achieves diagnostic accuracy comparable to or exceeding that of dermatologists for specific, well-defined tasks.

#### 1.1.1 Comparison with experienced specialists

Multiple studies confirm that AI accuracy is comparable to, and sometimes superior to, that of experienced dermatologists in diagnosing common and critical conditions such as skin cancer (particularly melanoma) (1–3). Some studies even conclude that AI may potentially outperform senior specialists (4). For instance, one study comparing a deep learning CNN model against 58 dermatologists demonstrated superior performance by the CNN in most cases (5). This high level of performance extends beyond melanoma; AI has also shown potential to outperform the majority of dermatologists in diagnosing other conditions, such as onychomycosis (6).

#### 1.1.2 Comparison with junior physicians

For early-career dermatologists, comparisons between AI and less experienced physicians are particularly relevant. Studies consistently demonstrate that AI significantly outperforms junior physicians or clinicians with limited experience in diagnostic accuracy (1, 7). A systematic review and meta-analysis explicitly indicates that AI can more effectively enhance the diagnostic performance of less experienced practitioners (1). The accuracy of AI in skin cancer detection is recognized as surpassing that of junior physicians, reaching levels comparable to specialists (4). This suggests AI can serve as a robust “safety net,” compensating for the experiential gaps inherent among early-career dermatologists.

#### 1.1.3 Potential for specific and rare diseases

AI capabilities are also extending into the diagnosis of specific, less common dermatological conditions. For instance, studies indicate that AI models can outperform participating dermatologists in the early diagnosis of mycosis fungoides (8). Concurrently, AI demonstrates potential in diagnosing autoimmune blistering diseases such as bullous pemphigoid and pemphigus (9, 10). However, it is important to note that no controlled trials have specifically compared the performance of AI to dermatology residents in diagnosing these rare diseases (11).

### 1.2 Current limitations and practical challenges

Despite AI’s impressive achievements, early-career dermatologists must maintain a clear understanding of its current limitations, which precisely underscore the enduring and irreplaceable value of human clinicians.

#### 1.2.1 The “black box” problem and lack of explainability

Many high-performance AI models, particularly deep learning networks, function as “black boxes,” where their decision-making process cannot be readily explained to clinicians and patients (12). This lack of transparency presents a major barrier to clinical adoption and the establishment of trust.

#### 1.2.2 Data bias and fairness concerns

The performance and generalizability of AI are highly dependent on the quality, diversity, and representativeness of its training datasets. A significant deficiency exists in most publicly available datasets regarding the representation of darker skin types (Fitzpatrick skin types V-VI), leading to substantially reduced diagnostic accuracy and potential algorithmic failure in these populations (13, 14). Furthermore, AI capabilities in diagnosing rare diseases are severely limited by the scarcity of sufficient training data (13). This lack of representation across the full spectrum of disease prevalence and skin types threatens to exacerbate existing healthcare disparities. Compounding these issues, many datasets lack geographic and ethnic diversity, as they are often curated from populations in high-income countries. An AI model trained on such a narrow dataset may fail to generalize effectively to patient populations from different geographic regions, ethnic backgrounds, or healthcare settings (15). This limited external validity represents a major barrier to the equitable global deployment of dermatological AI. Additionally, many publicly available datasets also suffer from simplistic binary labeling, which fails to capture the spectrum of skin diseases encountered in practice.

#### 1.2.3 The laboratory-real world gap (dataset shift)

A critical limitation of the current AI evidence base is that reported high-performance metrics often stem from studies utilizing meticulously curated and standardized image datasets. These idealized datasets are markedly distinct from the complex realities of routine clinical practice, where image quality is highly variable and artifacts (e.g., hair, skin markers, reflections, uneven lighting) are common (1, 15). This discrepancy, known as “dataset shift,” frequently leads to a degradation of AI performance when models are deployed in daily workflows. Consequently, the high accuracy rates reported in controlled laboratory studies may not translate directly to the clinic. This performance gap underscores the critical need for more prospective validation studies conducted within actual clinical workflows and across diverse practice settings to rigorously assess real-world efficacy and integration (16).

#### 1.2.4 Lack of holistic patient assessment

It is important to note that most AI validation studies are conducted on a per-lesion basis using isolated dermoscopic or clinical images. In contrast, dermatologists routinely perform a holistic patient assessment, evaluating the distribution of multiple lesions, identifying “outlier” lesions that deviate from the patient’s typical pattern, and integrating contextual clinical information. This comprehensive, patient-centered approach remains a significant challenge for current image-based AI systems, which are typically trained and validated on single-image tasks. Consequently, reported accuracy rates may overestimate AI-human equivalence in real-world clinical workflows where contextual and multi-lesion analysis is essential.

#### 1.2.5 Insufficient generalizability

An AI model trained on a specific dataset may fail to generalize effectively to images acquired from different geographic regions, diverse populations, or using different equipment (15). Furthermore, the absence of standardized testing protocols and validation against histopathological gold standards hinders the robust evaluation and comparison of different AI tools’ performance (17).

Many studies reporting high AI accuracy are based on binary classification tasks (e.g., melanoma vs. nevus), which do not reflect the complexity of real-world dermatologic diagnosis involving multiple differentials (18). While such systems demonstrate efficacy in narrow tasks, their performance drops significantly in multi-class settings or when faced with atypical presentations (6). This oversimplification risks misleading clinicians about AI’s readiness for broad clinical integration, underscoring the need for studies that evaluate AI in diagnostically challenging, multi-category scenarios.

## 2 The impact of artificial intelligence on the professional development of early-career dermatologists

The rise of AI is fundamentally reshaping the career trajectories of dermatologists. For early-career dermatologists, in particular, its influence spans all aspects of professional growth, from skill development to future professional roles.

### 2.1 Impact and transformation of clinical skill development

Residency training constitutes the critical period for the formation of clinical reasoning and diagnostic skills. The integration of AI within this process presents a dual impact.

#### 2.1.1 Risk of “de-skilling”

Over-reliance on AI diagnostic suggestions may trigger “automation bias” (19), characterized by the uncritical acceptance of AI outputs. This could potentially undermine the development of independent diagnostic thinking, pattern recognition, and intuitive clinical reasoning abilities in early-career dermatologists—a process traditionally cultivated through iterative practice, error-making, and reflection—posing a significant risk of “de-skilling” (20). Moreover, most AI systems are trained and validated on binary or limited-class datasets, which may not prepare trainees for the nuanced differential diagnoses required in complex cases. Over-reliance on AI could therefore impair the development of diagnostic skills for rare, atypical, or multi-morphological conditions. To mitigate this risk, residency training programs must incorporate deliberate pedagogical safeguards. These could include structured diagnostic exercises where trainees are required to formulate and justify a differential diagnosis *before* consulting AI outputs, fostering the development of independent clinical reasoning as a foundational skill (21, 22).

#### 2.1.2 Opportunity as a powerful educational tool

Conversely, AI also holds promise as a revolutionary educational tool (21). It can provide residents with access to a virtual database encompassing vast numbers of cases, offering immediate diagnostic feedback and comparative analysis. AI can personalize learning pathways by recommending targeted cases to reinforce knowledge in areas of weakness and may even facilitate real-time assessment of clinical milestone achievement (23). However, it is important to note that longitudinal studies investigating the long-term impact of AI tools on the development of residents’ diagnostic skills remain scarce (22). Furthermore, globally, concrete examples of systematic integration of AI into dermatology residency curricula are exceedingly rare, with few detailed implementation models publicly reported, whether in China, Japan, or South Korea (24).

### 2.2 Redefining the dermatologist’s role: the shift to AI-augmented workflows

**Figure 2:**
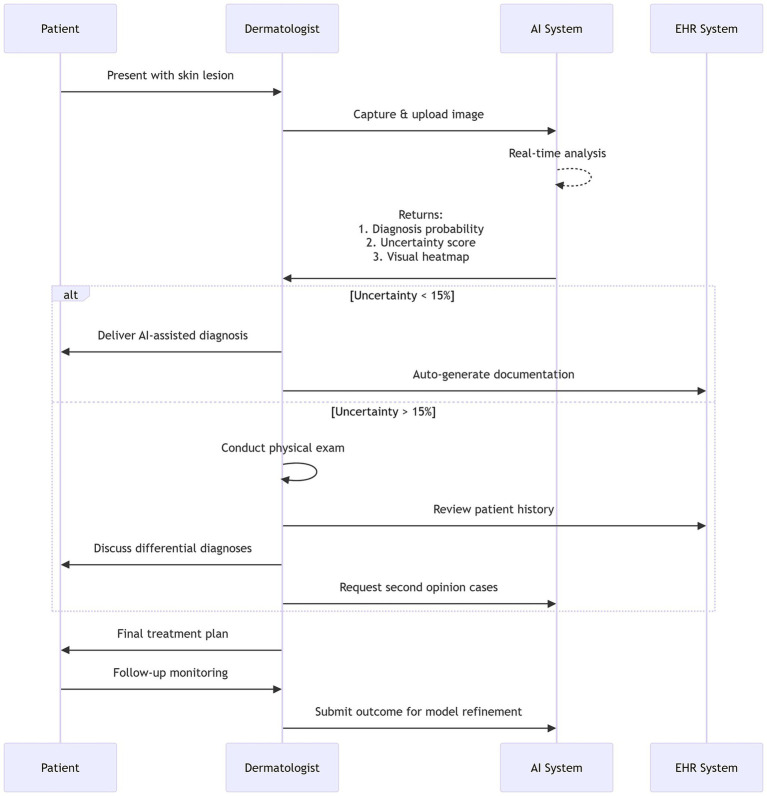
Human-AI collaborative workflow in dermatology practice.

The integration of AI is poised to fundamentally augment the dermatologist’s role by catalyzing a transition from performing repetitive, pattern-based tasks (e.g., initial lesion screening) toward focusing on higher-order cognitive and procedural functions. This evolution gives rise to the “human-AI collaboration” model, wherein the physician acts as the ultimate decision-maker. In this capacity, the dermatologist synthesizes AI-derived quantitative analyses with the patient’s comprehensive medical history, physical exam findings, and personal clinical experience to formulate a holistic judgment (Figure 2) (11, 25–27). This model effectively redefines the clinical workflow, positioning AI as a powerful diagnostic adjunct rather than a replacement.

However, the design of efficient and seamless collaborative workflows remains an open challenge, with limited documented evidence of standardized implementations in major academic centers (27, 28). Beyond diagnostic support, AI holds significant potential for workflow optimization and burnout mitigation. For instance, AI-powered digital scribes can automate medical note generation, substantially reducing administrative burdens and allowing physicians to dedicate more time to direct patient care and complex decision-making (29).

### 2.3 Anxiety and reality regarding “job displacement”

The question “Will AI replace dermatologists?” is an inescapable one for every young physician.

#### 2.3.1 Pervasive concerns

Surveys indicate widespread concern among healthcare professionals regarding the potential impact of AI on employment (30, 31). This apprehension also exists among dermatologists, albeit to varying degrees (32).

#### 2.3.2 Rational realistic assessment

However, the overwhelming majority of dermatologists express optimism or hold positive views toward AI, perceiving it as a powerful tool to augment diagnosis and treatment, rather than a replacement (33). The core value of dermatology extends far beyond image recognition. Empathy, humanistic care, building trusting relationships with patients, complex clinical reasoning (integrating history, signs, and laboratory findings), and mastery of procedural skills (such as biopsies, dermatologic surgery, and laser and cosmetic procedures) represent domains currently beyond the reach of AI. Consequently, comprehensive job displacement remains unrealistic in the foreseeable future.

## 3 Ethical, legal, and regulatory challenges in practice

Translating AI tools from the laboratory to the clinical setting requires young physicians to navigate a complex array of ethical, legal, and regulatory issues. The inherent uncertainties surrounding these multifaceted challenges represent a major barrier to the widespread adoption of AI in current healthcare practice.

### 3.1 Accountability: candidate frameworks and gaps in professional guidance

The integration of AI into diagnostic workflows introduces profound challenges in assigning accountability, moving beyond mere technical description to a core ethical and legal imperative for the profession.

#### 3.1.1 The complexity of causality and lagging legal frameworks

When an AI-assisted diagnosis proves erroneous and results in patient harm, attributing liability becomes a complex issue involving multiple stakeholders: the clinician responsible for the final decision, the hospital or healthcare institution that deployed and credentialed the tool, the algorithm developer, and potentially the data provider (34, 35). Current legal frameworks governing medical malpractice and product liability are proving inadequate to address these novel challenges (36–38). The “black-box” nature of many AI systems makes tracing the root cause of an error exceptionally difficult, further complicating liability determination (12).

#### 3.1.2 Candidate accountability frameworks

In response to this ambiguity, scholars and policy bodies are proposing candidate frameworks to distribute responsibility. A prominent model advocates for a shared or distributed responsibility framework (12, 34, 39). Under this model: Clinicians retain ultimate responsibility for the patient’s care and must exercise independent judgment in interpreting and acting upon AI outputs, adhering to a standard of “meaningful human oversight” (40). Healthcare Institutions are responsible for the rigorous validation, appropriate deployment, and continuous monitoring of AI tools within their clinical workflows, ensuring they meet standards of safety and equity. Developers and manufacturers are liable for the safety, efficacy, and transparent performance of their products under principles of product liability, which must be adapted for software that evolves over time (“learning” AI).

#### 3.1.3 Critical gaps in professional guidance

Despite the urgency of these issues, a significant regulatory and guidance vacuum persists. As of 2025, major international dermatology organizations, including the American Academy of Dermatology (AAD), have not issued official position statements or ethical guidelines specifying mechanisms for allocating liability in cases of AI diagnostic errors (41). This gap extends to a lack of clear directives regarding the circumstances under which physicians may or should override AI recommendations, as well as the legal liability thresholds associated with such actions (40–42). The absence of professional standards creates significant uncertainty for clinicians and underscores the critical need for dermatological societies to proactively shape ethical and legal norms for the AI-augmented era.

### 3.2 Informed consent and patient relationships

The integration of AI into clinical practice necessitates significant adaptations to the traditional informed consent process.

#### 3.2.1 Challenges to informed consent

Effectively explaining AI algorithms to patients poses substantial difficulties, particularly when both patients and physicians may lack a comprehensive understanding of the algorithms’ underlying mechanisms. Key concerns include elucidating potential biases, error rates, and data privacy risks inherent in AI systems. This complexity represents a critical ethical and communicative challenge in contemporary healthcare (43–45).

#### 3.2.2 Patient acceptance and trust

Empirical studies offer crucial insights into patient perspectives. Most patients express greater trust in human clinicians than in AI alone, with a strong preference for a collaborative “clinician-AI” model. Factors influencing patient acceptance include the perceived accuracy of AI, clinicians’ ability to interpret and endorse AI recommendations, and patient demographics (e.g., age, educational background) (46–48). These findings underscore the pivotal role of clinicians in facilitating human-AI interaction: they must communicate effectively with patients, manage expectations, and guide shared decision-making. Consequently, training early-career physicians in these competencies is essential to harmonize AI integration with patient-centered care.

### 3.3 Regulatory framework: navigating the approval landscape

As Software as a Medical Device (SaMD), AI-based tools undergo rigorous evaluation by regulatory authorities prior to market entry and clinical implementation.

#### 3.3.1 Regulatory pathways in major economies

In the United States, the U.S. Food and Drug Administration (FDA) regulates AI/ML-enabled medical devices through pathways including 510(k), *De Novo* classification, and Premarket Approval (PMA), having cleared 100 of such devices to date (49, 50). In the European Union (EU), AI medical devices must comply with the Medical Device Regulation (MDR) and the fully implemented Artificial Intelligence Act (EU AI Act). The latter imposes stringent requirements on high-risk AI systems, encompassing most diagnostic AI applications in medicine (51). While China demonstrates rapid advancement in AI healthcare, specific regulatory approval pathways for dermatological diagnostic tools remain undocumented in the available literature. Clinical imperative: Clinicians must verify regulatory clearance of any AI tool within their jurisdiction prior to clinical adoption.

## 4 Future-readiness strategy: strategic pathways for early-career dermatologists

Amid the transformative opportunities and challenges posed by AI in dermatology, neither passive hesitation nor apprehension will suffice. Early-career dermatologists must adopt a proactive stance—one defined by intentional skill development, ethical leadership, and systems-aware innovation—to position themselves as adaptable clinician-leaders poised to shape the future of the field (Table 1).

**Table 1:** Actionable framework for early-career dermatologists: addressing AI integration challenges.

Challenge category	Strategic response and best practices	Implementation timeline	Potential barriers and mitigation
Diagnostic skill development	Practice: Implement “AI-off” diagnostic simulation exercises (e.g., analyzing 10 dermoscopic images without AI aid) prior to reviewing AI output Tool: Use AI as a comparative feedback tool in tumor boards to discuss diagnostic discrepancies	Short-term (Ongoing): Integrate into weekly case reviews Long-term: Develop standardized AI-meditated assessment milestones	Barrier: Time constraints in residency Mitigation: Advocate for protected educational time and incorporate into existing academic activities
Workflow integration	Best Practice: Pilot a “AI Coordinator” role in outpatient clinics to manage AI tool use and data entry Example: Implement a protocol where AI triages low-complexity cases (e.g., seborrheic keratoses) to optimize scheduling	Short-term (6–12 mo): Design and pilot a protocol in one clinic Long-term (2+ yrs): Scale successful protocols department-wide	Barrier: Lack of IT support and EHR integration costs Mitigation: Start with standalone web-based AI tools; present cost–benefit analyses of time savings to institutional leadership
Legal accountability	Best Practice: Advocate for and adopt a shared responsibility framework (clinician-institution-developer) based on EU AI Act principles Action: Document rationale for overriding AI recommendations in the EHR	Immediate: Individual vigilance and documentation Long-term: Professional society advocacy for clear guidelines	Barrier: Ambiguous legal frameworks and fear of liability Mitigation: Institutional legal departments must develop AI use policies; use only regulatory-approved (e.g., FDA, CE) tools
Technical literacy	Concrete Example: Complete a short course on interpreting ROC curves and confusion matrices (e.g., Coursera’s “AI For Everyone”) Best Practice: Apply the CLEAR Derm checklist to critically evaluate one new AI study per month	Short-term (Within 3 mo): Complete foundational training Long-term (Ongoing): Stay updated on new model validations	Barrier: Perceived complexity and lack of formal training Mitigation: Petition residency programs to include mandatory AI literacy workshops; form journal clubs focused on AI critical appraisal
Patient communication	Best Practice: Develop and use specialty-specific consent templates that explain AI’s role, limitations, and data privacy measures Script: “I’m using an AI tool to help analyze your spot, which acts like a second opinion I’ll combine its analysis with my own expertise to make the best decision for you”	Short-term (Within 6 mo): Develop and adopt a clinic-wide communication template Long-term: Integrate templates into the EHR for streamlined use	Barrier: Patient skepticism and added time for explanations Mitigation: Train staff to handle common questions; use patient information leaflets to reinforce verbal communication
Regulatory navigation	Action: Create an institutional checklist for vetting AI tools (e.g., “Is this tool FDA-cleared for this specific use? What was the diversity of its training data?”) Resource: Rely on regulatory body databases (FDA, EUDAMED) for approval status	Pre-implementation: Mandatory compliance check for any new tool Ongoing: Monitor for post-market safety updates	Barrier: Rapidly evolving regulatory landscape Mitigation: Appoint an “AI Champion” within the department to monitor regulatory changes and disseminate updates

### 4.1 Embracing technology: becoming an “AI-augmented” physician

#### 4.1.1 Cultivating AI literacy

Young physicians should proactively acquire foundational knowledge of AI. While not required to become programmers, they need to understand the basic principles of machine learning, the critical importance of training data sets, the meaning of performance evaluation metrics (such as sensitivity, specificity, and AUC curve), and the sources and impact of algorithmic bias (52). This “AI literacy” will be a core competency for future physicians. Models such as AI workshops for residents in other disciplines (e.g., radiology) can be adapted to promote the inclusion of similar courses within dermatology training programs (53).

#### 4.1.2 Developing skills to critically appraise ai tools

Faced with a proliferation of AI products on the market, physicians require the ability to critically appraise their scientific validity and clinical utility. This includes reviewing the quality of validation studies, the representativeness of training data, the applicability of binary vs. multi-class diagnostic settings, real-world performance, and regulatory approval status. Guidelines such as the Checklist for Evaluation of Image-Based Artificial Intelligence Reports in Dermatology (CLEAR Derm) provide a valuable framework for assessing relevant studies (54–56).

### 4.2 Focusing on “human” advantages: strengthening irreplaceable core skills

While AI excels at pattern recognition, young physicians should intensify their focus on domains difficult for AI to replicate, thereby establishing a distinct competitive advantage (“moat”).

#### 4.2.1 Deepen comprehensive clinical diagnostic and management skills

Strengthen diagnostic and therapeutic capabilities for complex, rare, and systemic-disease-associated dermatological manifestations. This necessitates profound pathophysiological understanding and integrated clinical reasoning that transcend mere pattern recognition.

#### 4.2.2 Refine procedural skills

Invasive and non-invasive procedural skills—such as dermatologic surgery, laser therapy, cosmetic injections, and dermatopathology biopsy techniques—remain irreplaceable by AI. Young physicians should strive to master and refine these procedural skills extensively during residency training and early career stages.

#### 4.2.3 Enhance communication and humanistic care abilities

Establishing patient trust, conducting effective communication, providing emotional support, and delivering humanistic care constitute the essence of medicine. While AI processes data, the physician must simultaneously function as a compassionate communicator and caregiver.

### 4.3 Shaping the future: from training to practice

Young physicians should not merely be passive recipients of technology but must actively participate in shaping the evolution of AI within dermatology.

#### 4.3.1 Advocate for training reform

Proactively advocate for and propose the inclusion of AI-focused lectures, workshops, or the integration of AI tools into routine image interpretation sessions and case discussions within their own training programs (21). Given the current scarcity of such practices globally, the active involvement of young physicians could serve as a catalyst for change.

#### 4.3.2 Engage in clinical research and validation

Actively participate in clinical validation studies for AI tools (57). As frontline clinicians, the feedback from young physicians is critical for optimizing algorithms, refining human-computer interaction interfaces, and designing AI systems that better align with clinical needs.

#### 4.3.3 Pioneer new human-AI collaboration workflows

As standardized workflows do not yet exist, young physicians have the unique opportunity to act as both explorers and definers. Within their departments, they can pilot the integration of validated AI tools into existing workflows on a small scale. Documenting and evaluating the impact on efficiency, diagnostic accuracy, and clinician-patient experiences will generate invaluable evidence for future broader implementation (58, 59).

### 4.4 Understanding the realities of technology integration: addressing EHR integration challenges

A significant discrepancy often exists between technological ideals and practical implementation. Young physicians must recognize that seamlessly integrating an AI tool into a hospital’s electronic health record (EHR) system (e.g., Epic in the United States) is an exceptionally complex and costly process (60, 61). This requires interdepartmental collaboration and robust IT support, and does not occur overnight. Recognizing this reality helps manage expectations regarding new technology adoption and enables more effective participation in departmental and hospital-level technology adoption and integration planning.

## 5 Conclusion

Artificial intelligence represents not a “terminator” heralding the obsolescence of the physician’s role in dermatology, but rather a powerful “force multiplier.” It is reshaping diagnostic paradigms, transforming workflows, and imposing new demands on physicians’ skill sets. For early-career dermatologists embarking on their careers, this presents significant challenges alongside an unprecedented historical opportunity.

The future will favor neither those who resist technology nor those who blindly depend on it. Victory will belong to the “AI-augmented” dermatologist—one who possesses a profound understanding of AI’s capabilities and limitations, demonstrates proficiency in synergistic collaboration with AI, and leverages this partnership to maximize the application of uniquely human, irreplaceable wisdom and compassion. Through proactive learning, a focus on core human strengths, and active participation in shaping the future, early-career dermatologists are fully equipped to navigate this technological wave, defining and leading dermatology into its next era of excellence.
